# Real-Time Gait Analysis of the Pelvis and Lower Limbs Using a Seven-Node IMU Network

**DOI:** 10.3390/s26092776

**Published:** 2026-04-29

**Authors:** Xiao Wang, Lin Wang, Liangyang Luo, Enlin Cai, Shuying Wang

**Affiliations:** 1 College of Electronics and Information, Qingdao University, Qingdao 266071, China; 2018206198@qdu.edu.cn (X.W.); wanglin78@qdu.edu.cn (L.W.); luoliangyang@qdu.edu.cn (L.L.); 2 Aerospace Information Technology University, Jinan 250200, China

**Keywords:** IMU, wearable sensors, gait analysis, gait parameters

## Abstract

To address limited segment coverage and integration drift in wearable inertial gait analysis, this work proposes a real-time multi-segment gait analysis method using seven MEMS-IMUs deployed on the pelvis and lower limbs. The method employs parameter adaptive nonlinear complementary filtering and foot-based event detection to calculate spatiotemporal parameters and joint angles. Validation against optical motion capture (OMC) showed sagittal joint angle RMSEs below 2.37°, pelvic angle RMSEs below 0.96°, and correlation coefficients above 0.89 during normal walking in healthy adults. Supported by real-time 3D skeletal visualization, the proposed system provides a low-cost and portable solution for quantitative gait assessment under controlled walking conditions, with potential for future rehabilitation monitoring after further clinical validation.

## 1. Introduction

Human gait contains rich kinematic, kinetic, and physiological information and provides an important basis for clinical assessment, rehabilitation evaluation, and movement monitoring [1,2,3]. Its practical value has been demonstrated across a wide range of lower-limb and neurological applications, including therapist-guided robotic gait assistance for foot drop, real-time gait event detection for prosthesis control, mobile gait assessment in neurological disorders, and quantitative comparison of pathological and healthy gait patterns [4,5,6,7]. As quantitative functional assessment becomes increasingly important in rehabilitation and musculoskeletal care, gait analysis has gained growing relevance as an objective tool for identifying movement abnormalities and monitoring therapeutic outcomes [6,7].

Optical motion capture (OMC) remains the reference standard for gait analysis because of its high accuracy and ability to provide detailed segmental kinematics [8]. However, its use in routine clinical and community settings is limited by high equipment cost, dedicated laboratory requirements, complex setup procedures, and environmental constraints [9]. In contrast, wearable inertial measurement units (IMUs) offer a portable and cost-effective alternative that is well suited to out-of-laboratory monitoring [10]. Over the past decade, wearable-sensor-based gait analysis has been extensively investigated, and validation studies as well as systematic reviews have shown that inertial systems can provide clinically meaningful spatiotemporal and kinematic measurements under appropriate conditions [10,11,12,13,14,15].

To balance measurement precision, portability, and wearing burden, a broad range of wearable gait-analysis strategies has been explored. Some studies have focused on localized or task-specific solutions, including biomechanical gait fusion using vision and wearable sensors [16], real-world gait-bout detection using wrist-based sensing [17], seven-phase gait-cycle division from shank angular velocity [18], low-cost wearable knee monitoring [19], and inertial-sensor-based characterization of gait smoothness in elderly individuals [20]. Other studies have addressed robust walking detection in real-life settings, gait dynamics during robotic walker-assisted locomotion, and feature-rich quantitative gait assessment using a small number of IMUs [21,22,23]. These approaches are effective for specific tasks, but they often prioritize particular events, descriptors, or local segments rather than synchronous characterization of the complete pelvis–lower-limb kinematic chain.

To further improve practicality, recent research has increasingly investigated reduced-sensor and reconstruction-oriented frameworks. These include multi-IMU synchronization methods for distributed wearable networks [24], upper-limb and limb-segment calibration strategies [25], physics-informed learning for lower-limb kinematic prediction with sparse sensors [26], sparse-IMU global motion estimation enhanced by physical constraints [27], kinematics-based sensory fusion [28], and artificial-neural-network-based estimation of gait kinematics and kinetics from inertial data [29]. At the same time, commercial and field-based IMU systems, such as Xsens-based lower-extremity measurement and sacral-sensor running kinematics analysis, have demonstrated promising reliability under practical conditions [30]. Nevertheless, although reduced-sensor or predictive approaches can decrease instrumentation complexity, they may still depend strongly on model assumptions, prior datasets, or indirect inference of missing segments, which can limit their ability to resolve individualized inter-segment coordination and compensatory movement patterns with the same completeness as direct multi-node measurement [28,29,30,31].

Despite these advances, accurate capture of the pelvis–lower-limb kinematic chain during walking remains challenging. Recent validation and meta-analytic studies have emphasized that measurement accuracy depends strongly on sensor placement, calibration quality, segment coverage, and the robustness of the underlying fusion algorithm [13,14,15]. From an anatomical kinematic perspective, gait arises from coordinated motion of the pelvis and bilateral thighs, shanks, and feet. Therefore, incomplete segment coverage may limit the direct characterization of inter-segment coordination and compensatory movement patterns during walking. In many wearable configurations, the pelvis is not explicitly and synchronously measured together with all major lower-limb segments, which can lead to fragmented motion information and incomplete quantification of gait compensation mechanisms [3,23,30]. Simplified configurations with fewer sensors often focus on partial gait metrics or reconstruct joint angles via inverse kinematics inference, which may introduce additional estimation bias in clinical assessment of pathological gait and compensatory motion [23,26,27,28,29]. In addition, dynamic walking imposes substantial challenges for inertial tracking because impact, transient acceleration, heading instability, and inter-node timing inconsistency can degrade orientation estimation and long-term stability unless synchronization and drift-suppression strategies are carefully designed [28,29,30,31]. Moreover, full-chain synchronous measurement facilitates more robust kinematic estimation by preserving proximal and distal constraints within the same framework: the pelvic IMU captures proximal segment motion, while foot-mounted IMUs support gait-event detection and ZUPT-based drift correction. Therefore, there remains a need for an integrated wearable system that combines complete pelvis–lower-limb segment coverage with robust real-time attitude estimation.

To address these issues, this study proposes a real-time three-dimensional gait analysis system based on a seven-node distributed MEMS-IMU network. By placing synchronized sensors on the pelvis, bilateral thighs, shanks, and feet, the proposed system directly measures the pelvis–lower-limb kinematic chain rather than reconstructing missing segments from sparse proxy nodes. Building on the nonlinear complementary filter framework [31], a phase-aware parameter-adaptive strategy is introduced to improve orientation estimation by reducing acceleration-induced disturbance during dynamic phases while strengthening drift compensation during relatively stable gait intervals. Combined with foot-based gait-event detection, spatiotemporal parameter estimation, and C#-based real-time 3D visualization, the system is designed to provide a low-cost and portable solution for quantitative gait assessment and rehabilitation monitoring. Validation against an OMC reference further suggests that the proposed framework can achieve promising accuracy for sagittal lower-limb kinematics and selected pelvic motions during controlled walking in healthy adults.

## 2. Methods

**Figure 1:**
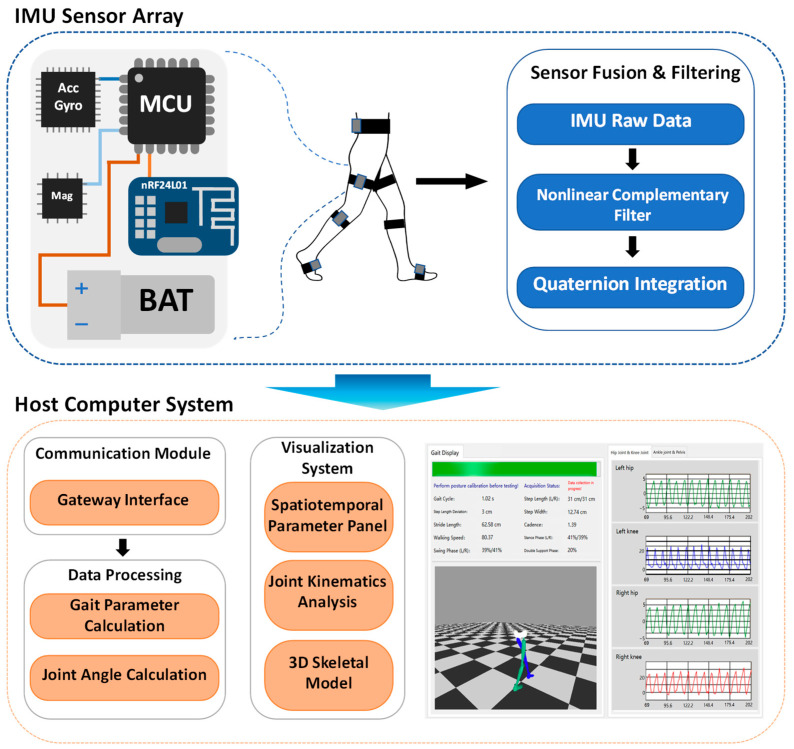
Architecture of the real-time 3D gait analysis system based on 7 IMUs.

This study constructed a real-time 3D gait analysis system based on a distributed multi-IMU node network. The core data processing flow encompasses: synchronized multi-node IMU data acquisition, spatial calibration from sensor coordinate systems to the anatomical coordinate system, real-time attitude estimation using nonlinear complementary filtering, detection of key gait events, calculation of spatiotemporal gait parameters and lower limb joint angles, and finally, real-time 3D visualization and analysis via PC software. The system architecture is shown in Figure 1.

### 2.1. Data Acquisition System

The hardware platform of this work is a distributed wireless sensor network consisting of one wireless receiving gateway and seven time-synchronized 9-axis MEMS IMU nodes, designed for full kinematic chain coverage of the pelvis and lower limbs to address spatial information fragmentation in sparse IMU-based gait systems.

Each IMU node is built around an STM32F405RGT6 microcontroller with a hardware floating-point unit, integrating an MPU-6050 6-axis inertial sensor and an HMC5983 triaxial magnetometer. The accelerometer is configured to a ±8 g full-scale range, the gyroscope to a ±1000°/s range, and all nodes operate at a unified sampling frequency of 200 Hz, matching the dynamic characteristics of normal human walking.

**Figure 2:**
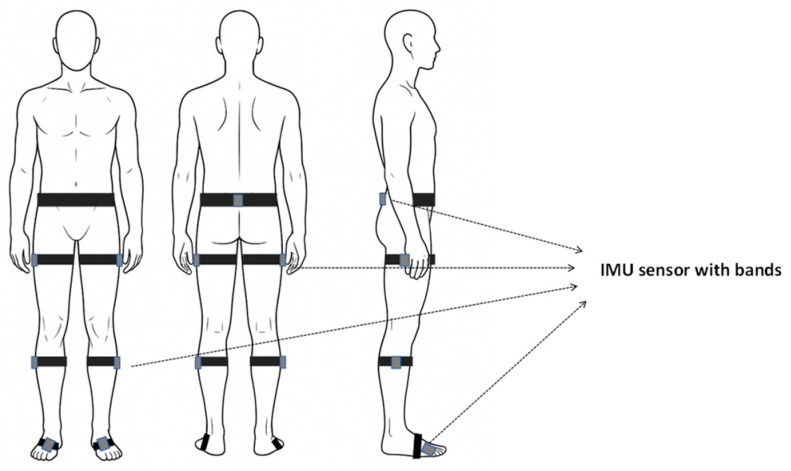
Schematic diagram of IMU sensor deployment locations. The seven IMUs were placed at the following anatomically defined sites: pelvis—L4–L5 level aligned with the bilateral anterior superior iliac spine direction; thighs and shanks—middle-to-distal third of each segment; feet—dorsum along the longitudinal axis of the foot.

As shown in Figure 2, the IMUs were secured to the pelvis and lower limbs using hypoallergenic elastic straps at anatomically meaningful locations: one pelvic node at the L4–L5 level aligned with the bilateral anterior superior iliac spine direction, and six limb nodes symmetrically attached to the middle-to-distal third of the bilateral thighs and shanks, as well as to the dorsum of both feet along the longitudinal axis of each segment. This seven-node configuration was selected to provide direct measurement coverage of the pelvis–lower-limb kinematic chain while maintaining acceptable wearing comfort and setup efficiency.

Before dynamic acquisition, each participant performed a standardized neutral standing posture for static initialization. This procedure was used to estimate the initial sensor-to-segment alignment and to define the transformation from the sensor coordinate system to the corresponding anatomical segment coordinate system. Although this procedure cannot completely eliminate placement-related errors, it reduces systematic misalignment introduced during sensor attachment and provides a consistent reference for subsequent segment orientation estimation.

In addition, all nodes underwent sensor calibration before each experiment, including gyroscope zero-bias estimation, six-position accelerometer calibration, and ellipsoid-based magnetometer calibration to compensate for hard- and soft-iron distortion. A three-stage cascaded preprocessing algorithm (clipping filter, sliding median filter, and sliding average filter) was implemented on-board to suppress outliers, reduce heel-strike-related impulse noise, and smooth residual fluctuations while preserving gait-event features.

To ensure temporal consistency across nodes, a master–slave synchronization scheme combining wireless broadcast synchronization and hardware-triggered sampling was implemented. The gateway acted as the master and transmitted synchronization beacons every 50 ms, while each slave node maintained local sampling using a 168 MHz hardware timer. Upon reception of each beacon, the slave node compared its local timer phase with the master reference and applied a phase correction to the subsequent sampling interval. This periodic phase correction compensated for long-term clock drift and prevented cumulative timing deviation during prolonged operation. Under laboratory conditions, the inter-node synchronization error remained within 1 ms.

Raw inertial data are transmitted to the host computer via a 2.4 GHz wireless protocol based on the nRF24L01+ chip, with a time division multiple access mechanism to support concurrent transmission from 7 nodes. The wireless link achieves an end-to-end latency ≤10 ms and a packet loss rate ≤0.1% via 16-bit cyclic redundancy check and automatic retransmission, providing reliable data input for real-time attitude estimation and 3D visualization.

To reduce motion artefacts during walking, the IMUs were attached at anatomically meaningful locations using elastic straps and aligned with the longitudinal axis of each segment as consistently as possible. During orientation estimation, the adaptive gain strategy further reduced the influence of transient acceleration disturbances during highly dynamic gait phases. Although these measures cannot completely eliminate soft-tissue motion or strap-related artefacts, they were intended to mitigate their influence on the measured signals and subsequent kinematic estimation.

The proposed prototype was implemented using commercially available MEMS-IMU components and embedded hardware. The approximate hardware cost of the full system was about CNY 2500, indicating a substantially lower hardware cost than laboratory-grade optical motion capture systems and many commercial motion-analysis platforms.

### 2.2. Real-Time Attitude Estimation and Gait Event Detection

Attitude calculation is performed in real-time on each node’s microcontroller to balance computational efficiency and precision. The system implements an orientation fusion framework based on the nonlinear complementary filter (NCF). The rigid body rotation is governed by the quaternion differential equation:

(1) $$\begin{array}{c} \dot{q}=\frac{1}{2}q⊗\left[\begin{array}{c} 0\\ \omega \end{array}\right] \end{array}$$

where ***ω*** = [*ω*_x_, *ω*_y_, *ω*_z_] ^T^ is the angular velocity vector measured by the gyroscope, ***q*** is the current quaternion, and ⊗ denotes quaternion multiplication.

To mitigate the cumulative integration drift inherent in tri-axial gyroscopes, a composite error vector ***e*** is synthesized through a spatial alignment process that fuses accelerometer and magnetometer data. This vector quantifies the angular mismatch between the observed external references and their estimated counterparts in the sensor frame:

(2) $$\begin{array}{c} e=({\hat{a}}_{m}\times {\hat{v}}_{g} \end{array})+\begin{array}{c} ({\hat{m}}_{m}\times {\hat{v}}_{b} \end{array})$$

where ${\hat{a}}_{m}$ is the normalized acceleration measurement, ${\hat{m}}_{m}$ is the normalized magnetometer measurement. ${\hat{v}}_{g}$ and ${\hat{v}}_{b}$ are the predicted reference vectors, and these are derived by projecting the global gravity vector $g^{n}$ and the magnetic reference $b^{n}$ from the navigation frame into the sensor frame using the current rotation matrix R(q).

The synthesized error ***e*** is then fed into a proportional-integral (PI) controller to generate the compensation term for the gyroscope:

(3) $$\begin{array}{c} \hat{\omega }=\omega_{gyro}-b+\begin{array}{c} \begin{array}{c} K_{p}e+K_{i}\int_{0}^{t}e\left(\tau \right)d\tau \end{array} \end{array} \end{array}$$

where $\omega_{\mathrm{gyro}}$ is the raw angular velocity measured by the gyroscope, ***b*** is the gyroscope bias, $K_{p}$, $K_{i}$ are the filter gains.

In contrast to a conventional NCF with fixed gains, this study adopted a phase-aware adaptive gain strategy to improve robustness during walking. Specifically, the proportional gain $K_{p}$ was adjusted according to the deviation of the acceleration magnitude from the gravitational constant, such that the influence of accelerometer-based correction was reduced during highly dynamic phases and increased when the measured acceleration was closer to quasi-static conditions:

(4) $$\begin{array}{c} K_{p} \end{array}=K_{p0}\cdot exp(-\alpha {\left(\left\|a\right\|-g\right)}^{2})$$

where $K_{p0}$ is the baseline proportional gain determined during static calibration, α is a shaping parameter that controls the sensitivity to acceleration disturbances, $\left\|a\right\|$ is the magnitude of the measured tri-axial acceleration, g is the standard gravity constant. Among them, the benchmark proportional gain $K_{p0}$ determined during the static calibration stage is 1.8, and the acceleration disturbance sensitivity shaping parameter α is 0.5. These parameters were calibrated through pre-experiments and can achieve a balance between the interference suppression during the high-dynamic swing phase and the steady-state accuracy during the stance phase.

**Figure 3:**
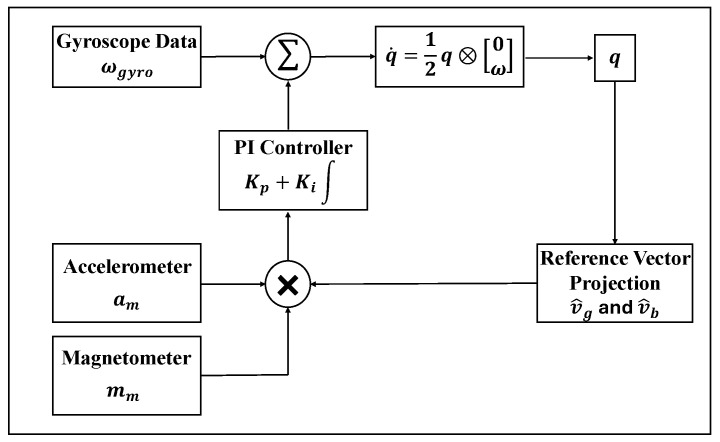
Block diagram of the attitude angle estimation algorithm.

The integral gain $K_{i}$, which mainly controls long-term drift compensation, was further modulated according to the detected gait phase. A larger integral correction was applied during stance-related intervals, when segment motion was relatively stable and the gravity reference was more reliable, whereas a smaller value was used during swing to reduce the risk of integrating transient dynamic disturbances into the bias estimate:

(5) $$\begin{array}{c} K_{i} \end{array}=\left\{\begin{array}{c} \begin{array}{c} K_{i,high},\;\;\;\;\;stance\;phase \end{array}\\ K_{i,low,}\;\;\;\;\;\;\;swing\;phase \end{array}\right.$$

the high-integration gain during the stance phase $K_{i,high}$ is set to 0.05, while the low-integration gain during the swing phase $K_{i,low,}$ is set to 0.005. The overall algorithm block diagram is shown in Figure 3.

Accurate identification of heel strike (HS) and toe off (TO) was essential for gait phase segmentation, spatiotemporal parameter extraction, and phase-dependent filter adaptation. In this study, gait events were detected from the foot-mounted IMU using a dual-feature strategy that combined vertical acceleration and sagittal-plane angular velocity. These two signals were selected because they provide complementary signatures of impact and rollover dynamics during normal walking.

Candidate HS and TO events were first identified from local extrema of the vertical acceleration signal within a short sliding window. To account for inter-subject variability in walking intensity, the detection thresholds were individualized using the mean peak and valley amplitudes observed during the first three stable gait cycles, multiplied by a scaling coefficient of 0.6. This value was chosen empirically as a conservative threshold that accommodates typical inter-subject variability in foot acceleration while minimizing false triggers from non-gait transients. A lower coefficient would increase sensitivity but risk spurious detections; a higher coefficient would improve specificity but could delay or miss low-amplitude heel strikes.

In a real-time scenario, the event-detection algorithm requires a short initialization period before individualized thresholds can be established. In this study, a gait cycle was regarded as stable when the temporal sequence of candidate heel-strike and toe-off events was physiologically consistent and the cycle duration showed no abrupt deviation relative to the immediately preceding cycle. After this initialization period, the individualized thresholds were used for subsequent real-time event detection, phase-dependent gain adaptation, and ZUPT correction. Thus, phase-adaptive operation was enabled after a short start-up phase rather than from the very first detected step.

To improve temporal specificity, each acceleration-based candidate event was further verified using the zero-crossing pattern of sagittal angular velocity within a ±50 ms temporal window. Only events satisfying both criteria were accepted as final HS or TO events. This logical conjunction improved the robustness of event detection by combining the impact sensitivity of acceleration with the directional transition information contained in foot angular velocity:

(6) $$\begin{array}{c} Event \end{array}=\left\{\begin{array}{l} \begin{array}{c} HS,\;\;\;\;\;\;\;{(a}_{y} \end{array}>peak)\cap {(\omega }_{z}\;crosses\;0\;↓)\\ \begin{array}{c} TO,\;\;\;\;\;{(a}_{y} \end{array}<valley)\cap {(\omega }_{z}\;crosses\;0\;↑) \end{array}\right.$$

where peak is the adaptive peak threshold for heel strike, valley is the adaptive valley threshold for toe off; *ω*_z_ crosses 0 ↓ indicates that the sagittal angular velocity crosses zero with a downward trend, and *ω*_z_ crosses 0 ↑ indicates that the sagittal angular velocity crosses zero with an upward trend.

### 2.3. Gait Parameter and Joint Angle Calculation

To reduce integration drift during foot trajectory reconstruction, a zero-velocity update (ZUPT) strategy was applied during the quasi-stationary portion of stance. Instead of assuming zero velocity throughout the entire interval between HS and TO, zero-velocity correction was imposed only within the foot-flat or mid-stance interval, during which the foot was assumed to move minimally relative to the ground. This design avoids over-constraining the trajectory near initial contact and toe off, where impact and push-off dynamics violate the zero-velocity assumption. After transforming the measured acceleration into the global frame and removing gravity, foot velocity and position were obtained by numerical integration with ZUPT-based drift correction.

Let ***P****_i_* = [*X_i_*, *Y_i_*, *Z_i_*]^T^ denote the reconstructed position of the foot-mounted sensor at the *i*-th initial contact. Based on these reconstructed trajectories and the detected gait events, the spatiotemporal gait parameters were calculated. Stride length was defined as the anteroposterior displacement between two successive initial contacts of the same foot in the global walking direction. Step width was defined as the mediolateral distance between contralateral foot contact points. Cadence was calculated from the number of completed steps per minute, and walking velocity was computed as the ratio between traveled distance and elapsed walking time:

(7) $$\begin{array}{c} SL= \end{array}{\left\|P_{IC,i+1}\right.\left.-P_{IC,i}\right\|}_{2}$$

(8) $$\begin{array}{c} SW=\left|X_{L,i}-X_{R,i}\right| \end{array}$$

(9) $$\begin{array}{c} C=\frac{n_{step}-1}{t_{end}-t_{start}} \end{array}\times 60,\begin{array}{c} V=\frac{∆Z}{∆t} \end{array}$$

where SL is the stride length (m); SW is the step width (m); C and V denote cadence (steps/min) and walking velocity (m/s) respectively.

Lower-limb joint kinematics were derived from the relative orientation between adjacent body segments within the hierarchical kinematic chain. To improve reproducibility and clinical interpretability, an anatomical coordinate system was assigned to each segment. The proximal–distal axis was taken as the longitudinal axis of the segment, the mediolateral axis represented the anatomical left–right direction of the segment, and the anteroposterior axis was defined accordingly to form a right-handed coordinate system. During the neutral standing calibration, an initial transformation between the IMU sensor frame and the corresponding anatomical segment frame was estimated and then used as the reference for subsequent segment orientation estimation. Joint angles were calculated from the relative orientation between adjacent segments and decomposed using a Z–Y–X Euler sequence. The Z–Y–X sequence was selected because it provided a clinically interpretable description of segment-to-segment rotation under the present anatomical frame definition, while allowing the primary sagittal-plane component to be extracted consistently for hip, knee, and ankle analysis. Under the present frame definition, the sagittal-plane rotational component of the relative orientation was used to represent hip flexion–extension, knee flexion–extension, and ankle dorsiflexion–plantarflexion, while pelvic tilt and pelvic rotation were obtained from the corresponding sagittal- and transverse-plane components of the pelvic segment orientation. Positive hip and knee angles indicated flexion, positive ankle angles indicated dorsiflexion, positive pelvic tilt indicated anterior tilt, and pelvic rotation was defined as the transverse-plane rotational component of the pelvic segment orientation, with its sign determined by the positive direction of the pelvic anatomical coordinate system. Because Euler-angle-based decomposition remains sensitive to axis definition and inter-plane coupling, some degree of cross-talk cannot be completely excluded. For each joint, the orientation of the distal segment was expressed relative to the proximal segment using quaternion-based coordinate transformation:

(10) $$\begin{array}{c} q_{joint}=q_{prox}^{-1}⊗q_{dist} \end{array}$$

In this model, $q_{prox}$ and $q_{dist}$ represent the orientations of the adjacent proximal and distal segments in the global frame. For clinical interpretability, the relative joint rotation was decomposed using a Z–Y–X Euler sequence. In the present study, primary analysis focused on sagittal-plane motion, including hip flexion–extension, knee flexion–extension, and ankle dorsiflexion–plantarflexion. Pelvic kinematics were quantified separately from the pelvic sensor orientation and included pelvic tilt and pelvic rotation. Although the system estimates three-dimensional segment orientations, the current validation focused mainly on sagittal lower-limb kinematics and selected pelvic motions, which are the most clinically relevant outputs for routine gait assessment in healthy walking. The sagittal plane motion *θ* is defined as:

(11) $$\begin{array}{c} \theta =atan2(2(q_{0}q_{1}+ \end{array}q_{2}q_{3}),1-2(q_{1}^{2}+q_{2}^{2}))$$

### 2.4. Architecture and Functional Implementation of C#-Based PC Gait Analysis System

The PC-side gait analysis software, developed in the C# environment, serves as the human–machine interaction and data processing hub of the system, providing multi-node synchronous data reception, gait kinematic calculation, quantitative assessment of walking patterns, and real-time 3D visualization. It converts raw inertial data into clinically interpretable gait metrics.

To ensure high-reliability data transmission from the 7 IMU nodes, the software adopts an event-driven asynchronous I/O model for serial communication with the wireless gateway. A multi-threaded architecture is designed to decouple high-frequency data reception, computationally intensive kinematic algorithm processing, and UI rendering, which avoids UI thread blocking and ensures the real-time responsiveness of the system during long-term continuous acquisition.

**Figure 4:**
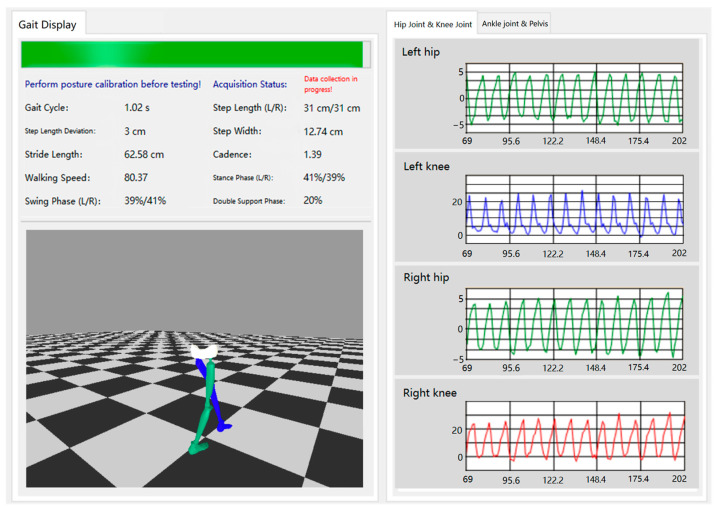
Visualization interface for gait parameters, skeletal model, and joint angles.

A high-performance non-blocking visualization engine is implemented to provide intuitive motion feedback for clinical assessment. The engine builds a 3D lower limb skeletal model matching human anatomical characteristics, with a parent–child hierarchical bone binding structure to restore the pelvis-lower limb kinematic chain. The main visualization interface of the system is shown in Figure 4.

In the proposed framework, raw inertial signals were synchronously sampled at 200 Hz on each IMU node. On-node processing included cascaded signal preprocessing and adaptive NCF-based orientation estimation, thereby reducing the computational burden on the host PC and enabling low-latency attitude updates at the sensor level. The host PC was responsible for wireless data reception, packet parsing, spatiotemporal and joint-angle calculation, real-time 3D visualization, and report generation.

At the system level, the proposed framework achieved an end-to-end latency below 40 ms, a host-side processing time below 5 ms per update cycle, and a visualization update rate of approximately 30–60 Hz. The estimated computational load on the MCU was approximately 30–50% during real-time operation, indicating that the proposed algorithm could be executed with sufficient processing margin on the embedded hardware. In addition, a preliminary pilot observation of repeated system operation under laboratory conditions showed stable operation over a cumulative duration of approximately 2 h, with no node disconnection, system crash, abnormal synchronization, or obvious overheating observed during the test sessions.

### 2.5. Optical Motion Capture Reference and IMU–OMC Alignment

To provide a reference for validation, gait kinematics were simultaneously measured using an eight-camera infrared optical motion capture system operating at 100 Hz. Reflective markers were attached to anatomical landmarks of the pelvis and lower limbs according to a standard lower-limb gait marker set, including bilateral anterior superior iliac spines, posterior superior iliac spines, thigh and shank tracking clusters, lateral femoral epicondyles, lateral malleoli, heel, and toe markers. Based on the marker trajectories, segment coordinate systems were reconstructed using a standard lower-limb biomechanical model. Hip, knee, and ankle joint angles, as well as pelvic tilt and rotation, were calculated from the relative orientation between adjacent segments. To maximize comparability, the rotational definitions of the IMU-based and OMC-based kinematics were matched as closely as possible, and relative segment rotations were expressed using the same Z–Y–X Euler decomposition convention for comparison. The primary analysis focused on sagittal-plane motion. Marker trajectories were filtered using a fourth-order zero-lag Butterworth low-pass filter with a cutoff frequency of 6 Hz. Temporal alignment was performed offline using a signal-based cross-correlation procedure based on pelvic-segment motion at the beginning of each trial, thereby avoiding circular dependence on the joint-angle variables under validation. After alignment, both datasets were resampled to a common 100 Hz time base using linear interpolation to enable point-by-point waveform comparison, RMSE calculation, and correlation analysis.

## 3. Experiment and Results

### 3.1. Experimental Protocol

To evaluate system performance, a comparative experiment was designed to validate joint angles and spatiotemporal gait parameters measured by the proposed system against those from an optical motion capture (OMC) system. The study was approved by the Ethics Committee of the Medical Department of QingDao University and followed the ethical principles of the Declaration of Helsinki. All participants provided written informed consent after receiving detailed explanations of the experimental procedures, potential risks, and benefits, and were assured of their right to withdraw at any time without penalty. Data were anonymized to protect privacy. Ten healthy adults (5 males, 5 females; age 22–26 years; height 160–185 cm; weight 55–100 kg) with no history of gait-affecting neurological or musculoskeletal disorders participated in the validation. Healthy adults with normal gait were selected as the validation population in this study because they provide a relatively standardized and repeatable benchmark for initial methodological validation of the system. This design was intended to evaluate the basic accuracy and stability of the proposed framework under controlled walking conditions before future validation in pathological gait with more complex, asymmetric, and non-periodic motion patterns. During installation, subjects maintained a static reference posture while IMUs were secured in anatomical order, followed by automatic sensor coordinate system calibration to compensate for individual anatomical variations. Reflective markers were then attached to key anatomical landmarks according to OMC system requirements, and system calibration was completed. For dynamic acquisition, subjects stood still for 2–3 s before walking along an approximately 20 m straight walkway at a self-selected comfortable speed using their natural gait. Each trial lasted approximately 15 s and comprised approximately 15 complete gait cycles. Upon completion of the walking task, subjects again stood still for 2–3 s, at which point the system automatically terminated data acquisition and saved timestamp-marked data for the complete motion cycle. This procedure was repeated ten times per subject to provide sufficient paired observations for statistical comparison under controlled walking conditions. All participants completed the experimental protocol without interruption or sensor detachment. In addition, informal verbal feedback was collected after the trials, and the participants generally reported acceptable short-term wearability without obvious interference with normal walking during the experimental sessions. However, no structured comfort questionnaire or formal usability assessment was included in the present study.

### 3.2. Results

To evaluate the performance of the proposed system, lower-limb sagittal kinematics and spatiotemporal gait parameters obtained from the IMU-based system were compared with those measured by the optical motion capture (OMC) system during normal walking. Ten healthy adults completed repeated walking trials at a self-selected comfortable speed. For the main kinematic variables, the agreement between the two systems was quantified using the root mean square error (RMSE), which reflects the average magnitude of measurement error, Pearson correlation coefficients, mean bias, 95% limits of agreement (LoA), and intraclass correlation coefficients (ICC), which reflect the consistency between the IMU-based measurements and the OMC reference. Bland–Altman analysis was further used to visualize systematic bias and dispersion between the IMU-based measurements and the OMC reference. The unit of analysis was the trial-level paired observation, with one IMU-derived value and one OMC-derived value for each variable in each trial.

Table 1 summarizes the spatiotemporal gait-parameter errors between the IMU and OMC systems. The OMC mean values are displayed in rounded form for readability; all error metrics were calculated from the original unrounded trial-level measurements. The RMSE was 0.167 ± 0.032 s for gait cycle, 0.0285 ± 0.004 m for step length, 0.0247 ± 0.005 m for stride length, and 0.030 ± 0.007 m/s for walking velocity. Using the mean OMC values as reference, the corresponding relative RMSE values were 15.9%, 4.2%, 1.8%, and 2.3%, respectively. Although the gait-cycle error was relatively higher in percentage terms, this should be interpreted together with its absolute error. Overall, the relatively small absolute and percentage errors for step length, stride length, and walking velocity indicate that the proposed system can provide stable estimates of basic spatiotemporal gait parameters under normal walking conditions.

The quantitative comparison of lower-limb joint angles is presented in Table 2. For sagittal-plane joint motion, the hip, knee, and ankle angles all achieved RMSE values below 3°, with corresponding correlation coefficients above 0.89. Specifically, the hip angle showed a correlation coefficient of 0.91 ± 0.03 and an RMSE of 2.18 ± 0.25°, the knee angle showed a correlation coefficient of 0.90 ± 0.03 and an RMSE of 2.21 ± 0.41°, and the ankle angle showed a correlation coefficient of 0.89 ± 0.03 and an RMSE of 2.37 ± 0.45°. These results demonstrate good agreement between the IMU-based system and the OMC reference for sagittal lower-limb kinematics throughout the gait cycle.

Pelvic motion was also evaluated to assess the system’s ability to capture proximal segment kinematics. Pelvic tilt and pelvic rotation both exhibited high correlations with the OMC reference, with correlation coefficients of 0.93 ± 0.03 and 0.92 ± 0.03, respectively. The pelvic variables also showed low errors, with RMSEs of 0.96 ± 0.18° for pelvic tilt and 0.96 ± 0.19° for pelvic rotation. Compared with the lower-limb joint angles, pelvic motion showed the smallest angular error, suggesting that the proposed seven-node configuration can provide stable estimation of pelvic orientation during walking.

**Figure 5:**
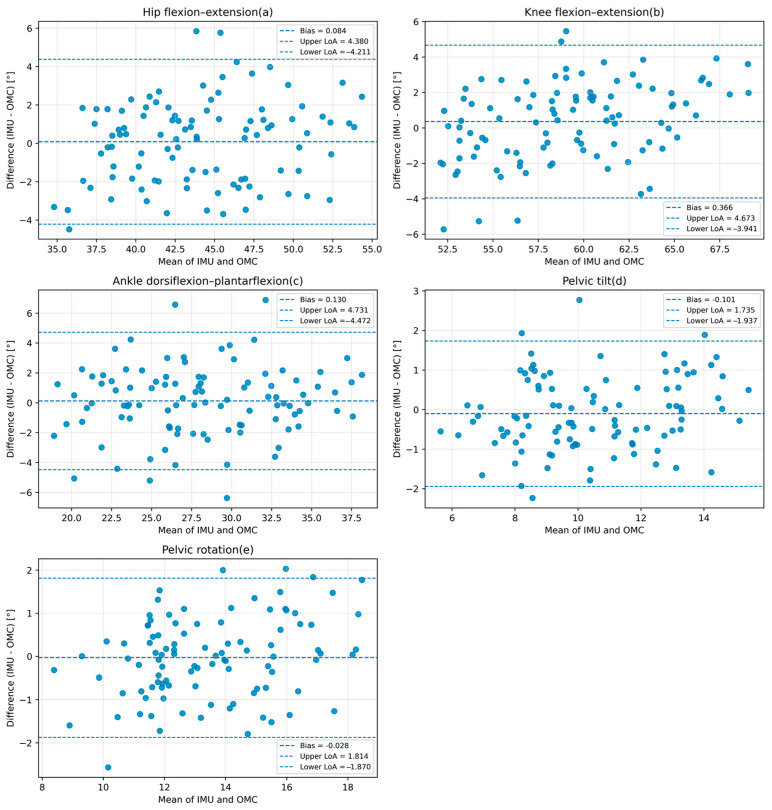
Bland–Altman plots for the main kinematic variables comparing the IMU-based system with the OMC reference. Panels show (**a**) hip flexion–extension, (**b**) knee flexion–extension, (**c**) ankle dorsiflexion–plantarflexion, (**d**) pelvic tilt, and (**e**) pelvic rotation.

In addition to RMSE and correlation coefficients, agreement-oriented statistics were calculated for the main kinematic variables. The corresponding Bland–Altman plots are presented in Figure 5, in which each point represents one trial-level paired observation, and the representative scalar for each variable was defined as the mean range of motion (ROM) across all valid gait cycles within that trial. The ICC values were 0.900 for hip angle, 0.881 for knee angle, 0.891 for ankle angle, 0.925 for pelvic tilt, and 0.921 for pelvic rotation, indicating good consistency between the IMU-based measurements and the OMC reference. The Bland–Altman results further showed that the mean bias values were small for all kinematic variables, indicating no obvious systematic offset between the two systems. Pelvic tilt and pelvic rotation showed relatively narrow limits of agreement, within ±2°, whereas hip, knee, and ankle motion showed somewhat wider limits of agreement, within ±4–5°. For the present controlled-condition validation in healthy adults, these limits of agreement were considered acceptable for sagittal lower-limb motion and the selected pelvic variables.

**Figure 6:**
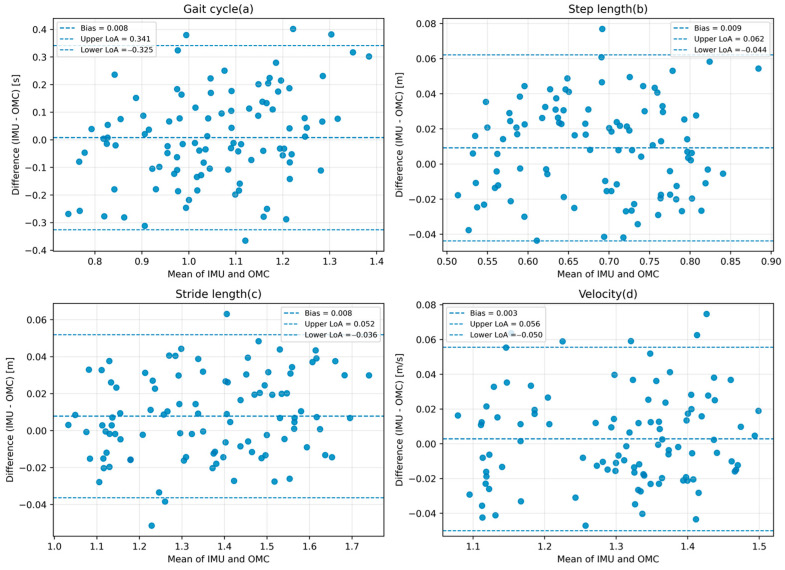
Bland–Altman plots for the main spatiotemporal gait parameters comparing the IMU-based system with the OMC reference. Panels show (**a**) gait cycle, (**b**) step length, (**c**) stride length, and (**d**) walking velocity.

Agreement analysis was also performed for the main spatiotemporal gait parameters using trial-level paired observations, and the corresponding Bland–Altman plots are presented in Figure 6. Step length, stride length, and walking velocity showed mean bias values close to zero and comparatively narrow limits of agreement, indicating favorable consistency with the OMC reference under normal walking conditions. In contrast, gait cycle showed a wider agreement range, suggesting greater variability between the two systems for this parameter. Because gait-cycle estimation depends directly on the timing of heel-strike and toe-off detection, even modest event-timing differences may have a more direct influence on this parameter than on some of the other spatiotemporal variables. Therefore, the limits of agreement for the spatiotemporal parameters were considered acceptable overall for the present controlled-condition validation, while gait cycle should be interpreted with greater caution than step length, stride length, and walking velocity.

To isolate the contribution of the proposed adaptive gain strategy, we additionally compared the parameter-adaptive NCF with a conventional fixed-gain NCF under the same seven-IMU configuration, calibration procedure, gait-event detection, and kinematic computation pipeline. The only difference between the two methods was whether the filter gains were adaptively adjusted during walking. The RMSE comparison is presented in Table 3.

As shown in Table 3, the proposed adaptive NCF reduced the RMSEs of most evaluated kinematic variables compared with the fixed-gain baseline, indicating that the adaptive gain strategy contributed to the overall performance improvement.

To verify that the adaptive filter parameters are not over-tuned to the specific walking conditions of the present dataset, a univariate sensitivity analysis was conducted. Each key parameter was perturbed by ±50% of its nominal value while all other parameters and the remaining processing pipeline were held unchanged. For each configuration, the mean root-mean-square error (RMSE) of the sagittal hip, knee, and ankle angles, as well as pelvic tilt and rotation, was recomputed using the ten-subject dataset. The sensitivity results are summarized in Table 4.

The maximum increase in mean RMSE under ±50% perturbation was 0.15° for the hip, 0.13° for the knee, 0.12° for the ankle, 0.03° for pelvic tilt, and 0.04° for pelvic rotation. These limited changes suggest that the selected parameter values were reasonably stable within the tested perturbation range.

**Figure 7:**
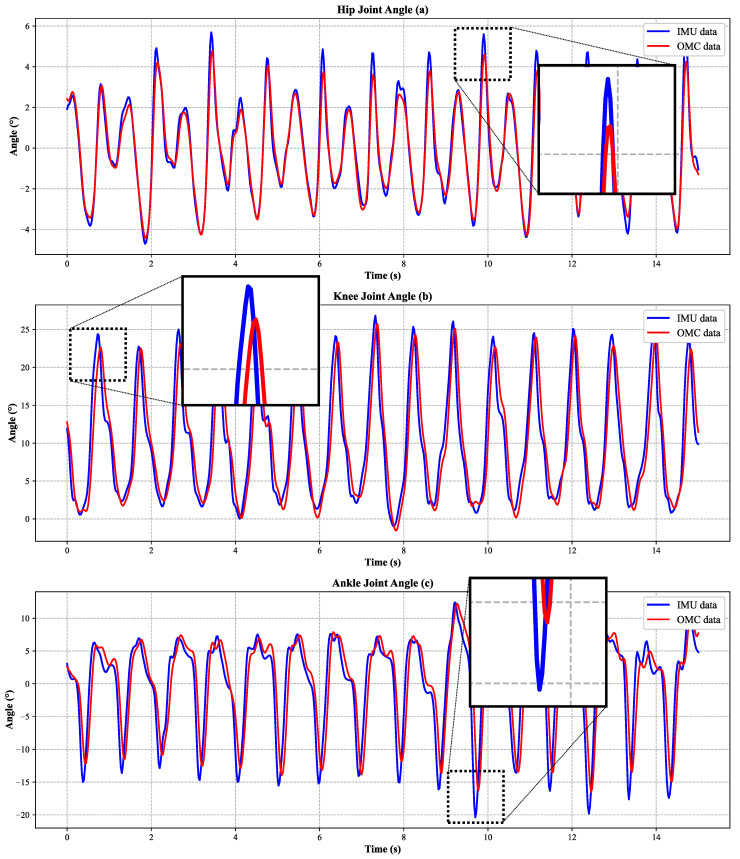
Comparison of sagittal-plane lower-limb joint angles measured by the proposed IMU system and the optical motion capture (OMC) system over the full gait cycle. Panels show (**a**) hip flexion-extension, (**b**) knee flexion-extension, and (**c**) ankle dorsiflexion-plantarflexion. The x-axis represents time (s), and the y-axis represents joint angle (°). Blue lines denote IMU measurements, and red lines denote OMC measurements. Zoomed insets highlight the regions of maximum discrepancy. The plots show one representative trial from one representative participant.

**Figure 8:**
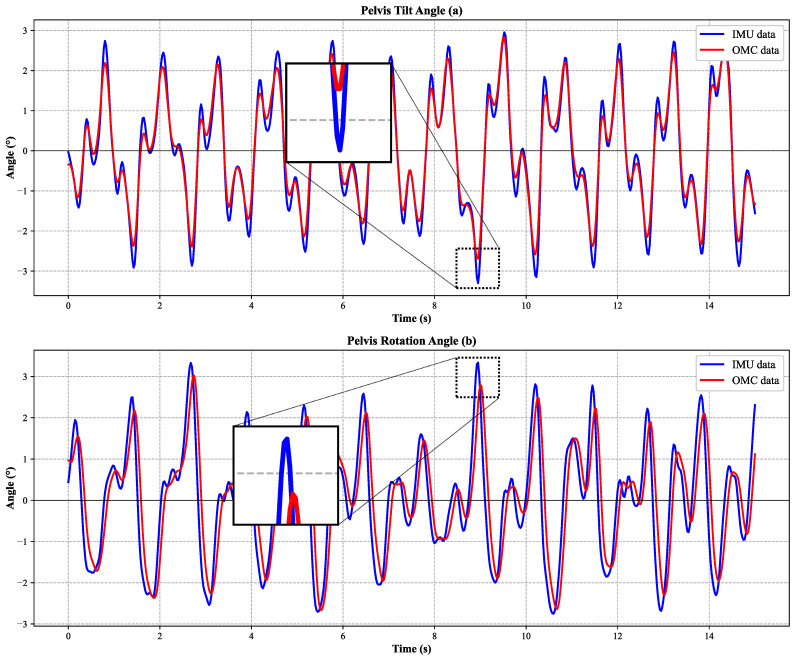
Comparison of pelvic kinematics measured by the proposed IMU system and the optical motion capture (OMC) system over the full gait cycle. Panels show (**a**) pelvic tilt and (**b**) pelvic rotation. The x-axis represents time (s), and the y-axis represents angle (°). Blue lines denote IMU measurements, and red lines denote OMC measurements. Zoomed insets highlight the regions of maximum discrepancy. The plots show one representative trial from one representative participant.

Figure 7 illustrates the full-gait-cycle trajectories of hip flexion–extension, knee flexion–extension, and ankle dorsiflexion–plantarflexion obtained from the IMU-based system and the OMC system. The two systems showed similar waveform trends across the gait cycle, indicating that the proposed method can reproduce the major temporal patterns of sagittal lower-limb motion. To make the enlarged local discrepancies more explicit, the maximum absolute differences within the zoomed regions of the representative trial were quantified as 2.44° for hip flexion–extension, 4.57° for knee flexion–extension, and 7.06° for ankle dorsiflexion–plantarflexion. These deviations were localized and transient rather than persistent over the full waveform. Among the three sagittal-plane joint variables, the ankle waveform showed the largest local discrepancy, whereas the hip waveform remained more closely matched to the OMC reference in the enlarged view. Figure 8 presents the corresponding comparison for pelvic tilt and pelvic rotation, and similarly demonstrates close agreement in waveform shape between the two measurement systems. The maximum absolute differences within the zoomed regions were 1.18° for pelvic tilt and 1.32° for pelvic rotation, indicating that the local discrepancies of the pelvic variables were smaller than those of the lower-limb joint angles. Overall, these enlarged local variations are relevant as examples of the largest transient waveform mismatch in the representative trial, but they do not alter the overall agreement trend between the IMU-based measurements and the OMC reference.

To further position the proposed framework relative to previous methods, Table 5 compares this work with representative gait analysis systems. The proposed system employs seven IMUs and a parameter-adaptive nonlinear complementary filter, achieving hip/knee/ankle angle errors of 2.18°/2.21°/2.37° and correlation coefficients of 0.91/0.90/0.89. In addition, unlike the two reference methods summarized in Table 5, the present system includes pelvic motion measurement. These results suggest that the proposed framework provides competitive lower-limb kinematic accuracy while extending the measurement scope to the pelvis. However, direct cross-study comparison should be interpreted cautiously because the validation protocols, populations, and error definitions differed across studies.

## 4. Discussion

This study developed and validated a real-time gait analysis system based on a seven-node IMU network for simultaneous measurement of lower-limb and pelvic kinematics during walking. Compared with the optical motion capture (OMC) reference, the proposed system achieved low errors in spatiotemporal gait parameters and maintained sagittal-plane joint angle RMSEs below 3°, while pelvic tilt and pelvic rotation errors remained below 1°. These findings support the feasibility of the proposed framework for portable quantitative gait analysis under controlled walking conditions.

One notable strength of the proposed system is its complete sensor coverage of the pelvis and lower limbs. By deploying IMUs on the pelvis, bilateral thighs, shanks, and feet, the system directly measures the major segments of the lower-limb kinematic chain rather than inferring missing motion from sparse sensing. This design is advantageous because pelvic motion plays an important role in gait compensation and whole-body coordination, yet it is not explicitly captured in many simplified IMU configurations. In the present study, pelvic tilt and pelvic rotation showed the smallest angular errors among all evaluated kinematic variables, with RMSE values of 0.96° and correlation coefficients above 0.92, which suggests that the additional pelvic node improves the completeness and stability of the kinematic reconstruction.

A second strength of this work lies in the phase-aware adaptive fusion framework. The proposed method extends the conventional nonlinear complementary filter by adjusting the filter gains according to acceleration consistency and gait phase. Because walking alternates between relatively stable stance periods and more dynamic swing periods, a fixed-gain strategy may not provide optimal performance throughout the entire gait cycle. The present results indicate that this adaptive design can effectively support orientation estimation during normal walking, as reflected by the favorable agreement with OMC in both lower-limb joint angles and pelvic kinematics. In particular, the fact that hip, knee, and ankle RMSE values were all below 3° suggests that the system can achieve promising accuracy for sagittal gait assessment under controlled walking conditions.

The spatiotemporal results further demonstrate the practical utility of the system. The RMSE values for step length, stride length, and walking velocity were 0.0285 m, 0.0247 m, and 0.03 m/s, respectively, indicating that the event-driven foot trajectory reconstruction pipeline can provide stable estimates of commonly used gait parameters. Since these parameters are commonly used in rehabilitation evaluation and longitudinal monitoring, the proposed system may be particularly suitable for repeated assessments in settings where laboratory-grade OMC is impractical due to cost, space, or operational complexity.

Despite these promising findings, several limitations should be acknowledged. First, the current study represents a methodological validation of the proposed 7-node IMU system, and the validation was conducted only in ten healthy young adults walking at a self-selected comfortable speed on level ground. Therefore, the present results reflect system performance under relatively regular gait conditions and may not generalize directly to pathological gait, variable speeds, turning, stair negotiation, or free-living environments. Pathological gait is typically characterized by greater inter-subject variability, asymmetry, and non-periodicity, which may place higher demands on algorithm robustness and measurement stability. Therefore, dedicated clinical validation is still required. Second, motion artefacts remain an important source of uncertainty in wearable IMU-based gait analysis. Although static standing calibration provides a practical and efficient initialization strategy, it cannot fully remove sensor-to-segment misalignment, strap micro-slippage, or soft-tissue oscillation during walking, especially on the thigh and shank segments. In the present study, standardized sensor placement, elastic fixation, cascaded preprocessing, and adaptive filtering were used to reduce these effects; however, their influence was not isolated in a dedicated quantitative experiment. Residual motion artefacts may therefore have contributed to the observed joint-angle errors. Future work will include repeated sensor reattachment tests and segment-specific analyses to further evaluate and reduce motion artefacts. In addition, the accuracy of heel-strike and toe-off detection was not independently validated against an external reference in the present study. Because gait-event detection affects both phase-dependent gain adaptation and ZUPT correction, dedicated validation of event-detection accuracy should be included in future work. Third, magnetometer measurements were incorporated to assist long-term orientation stabilization, but magnetic disturbances in indoor environments may still affect heading estimation. Finally, although the system estimates three-dimensional segment orientations, the present validation focused mainly on sagittal lower-limb motion and selected pelvic variables, and additional validation in the frontal and transverse planes is still needed.

The current study also suggests several directions for future work. A key priority is to extend system validation to clinical populations with gait disorders, such as post-stroke hemiplegia, Parkinson’s disease, knee osteoarthritis, and spinal cord injury. Future clinical validation will require appropriate ethical approval, clearly defined inclusion and exclusion criteria, and multidimensional evaluation endpoints, including the accuracy of kinematic measurements, agreement with clinical functional assessments, and reliability in longitudinal rehabilitation monitoring. In practical rehabilitation settings, the proposed system may support clinicians by generating quantitative gait analysis reports that include commonly used spatiotemporal parameters and lower-limb/pelvic kinematic variables. These outputs may help identify abnormal gait patterns, quantify asymmetry and compensatory strategies, and compare patient performance across repeated treatment sessions. Based on this reporting framework, future versions of the system may further incorporate therapist-oriented feedback functions, such as visual highlighting of abnormal gait indicators, auditory cues when selected parameters deviate from predefined target ranges, or phase-specific vibrotactile cues to facilitate gait retraining. Moreover, incorporating more robust sensor-to-segment calibration and magnetic disturbance rejection strategies may improve accuracy in more challenging locomotor tasks and non-laboratory environments. Furthermore, the present study was limited to self-selected comfortable walking over approximately 15 s trials. Dedicated assessment of cumulative integration drift across a range of walking speeds is still needed to further evaluate system robustness under more varied dynamic conditions. In addition, although a preliminary pilot observation of repeated system operation under laboratory conditions showed no node disconnection, system crash, abnormal synchronization, or obvious overheating during a cumulative operating duration of approximately 2 h, battery endurance, packet-loss statistics, and drift accumulation were not systematically quantified. Therefore, dedicated long-duration continuous-use evaluation is still needed. Although the gait-event detection strategy was used to support phase-dependent gain adaptation and ZUPT correction, a dedicated validation of heel-strike and toe-off detection accuracy against an external reference was not included in the present study. This remains an important topic for future work. With these improvements, the proposed seven-node IMU system may become a practical tool for portable gait screening, rehabilitation monitoring, and routine quantitative assessment in resource-limited clinical settings.

## 5. Conclusions

In summary, this study proposed a real-time gait analysis framework based on a seven-node IMU network and demonstrated promising agreement with the OMC reference for spatiotemporal parameters, sagittal lower-limb kinematics, and selected pelvic motions during controlled walking in healthy adults. These findings support the feasibility of the proposed system as a portable tool for quantitative gait analysis under controlled conditions. However, further validation in clinical populations, more diverse locomotor tasks, and additional frontal/transverse plane analyses are still required before broader applications in rehabilitation monitoring, pathological gait assessment, and practical real-world deployment can be established.

## Figures and Tables

**Table 1:** Comparison of spatiotemporal gait-parameter errors between the IMU and OMC systems under normal walking conditions.

Parameter	Gait Cycle (s)	Step Length (m)	Stride Length (m)	Velocity (m/s)
OMC mean	1.05	0.68	1.36	1.30
RMSE	0.167 ± 0.032	0.0285 ± 0.004	0.0247 ± 0.005	0.030 ± 0.007
Relative RMSE(%)	15.9	4.2	1.8	2.3

**Table 2:** Agreement metrics of joint-angle variables between the IMU and OMC systems under normal walking conditions.

Joint	Correlation Coefficient	RMSE (°)	ICC
Hip	0.91 ± 0.03	2.18 ± 0.25	0.900
Knee	0.90 ± 0.03	2.21 ± 0.41	0.881
Ankle	0.89 ± 0.03	2.37 ± 0.45	0.891
Pelvis tilt	0.93 ± 0.03	0.96 ± 0.18	0.925
Pelvis rotation	0.92 ± 0.03	0.96 ± 0.19	0.921

**Table 3:** RMSE comparison between the proposed adaptive NCF and a conventional fixed-gain NCF under the same experimental conditions.

Method	Hip RMSE (°)	Knee RMSE (°)	Ankle RMSE (°)	Pelvic Tilt RMSE (°)	Pelvic Rotation RMSE (°)
Fixed-gain NCF	2.42	2.56	2.74	1.05	1.07
Proposed adaptive NCF	2.18	2.21	2.37	0.96	0.96

**Table 4:** Sensitivity of the proposed adaptive NCF to variations in key filter parameters.

Configuration	Hip RMSE (°)	Knee RMSE (°)	Ankle RMSE (°)	Pelvic Tilt RMSE (°)	Pelvic Rotation RMSE (°)
Baseline (*K*_p0_ = 1.8, *α* = 0.5)	2.18	2.21	2.37	0.96	0.96
*K*_p0_ + 50% (2.70)	2.24	2.31	2.46	0.98	0.97
*K*_p0_ − 50% (0.90)	2.33	2.34	2.49	0.99	1.00
*α* + 50% (0.75)	2.20	2.23	2.41	0.97	0.98
*α* − 50% (0.25)	2.22	2.27	2.38	0.98	0.97

**Table 5:** Comparison of system performance with representative methods.

	This Work	[28]	[29]
Number of IMUs	7	7	5
Methods	Parameter-adaptive NCF	EKF	ANN
Joint Angle Error	2.18/2.21/2.37	<5/<5/<5	2.42/4.62/—
Correlation coefficient	0.91/0.90/0.89	0.89/0.97/0.99	>0.85/>0.85/>0.85
Pelvic motion measured	Yes	No	No

## Data Availability

The data presented in this study are not publicly available due to privacy and ethical restrictions related to human-subject data.

## Abbreviations

The following abbreviations are used in this manuscript:

IMU	Inertial Measurement Unit
OMC	Optical Motion Capture
MEMS	Micro-Electro-Mechanical System
NCF	Nonlinear Complementary Filter
PI	Proportional-Integral
HS	Heel Strike
TO	Toe Off
ZUPT	Zero-Velocity Update
RMSE	Root Mean Square Error
